# “Cold” on the Hear

**Published:** 1888-03

**Authors:** A. Luton, F. R. Campbell

**Affiliations:** Professor of Clinical Medicine at Reims


### Translations.

### "COLD" ON THE HEART.

(*Le Rhume du Cœur.*)

By A. LUTON, Professor of Clinical Medicine at Reims.

Translated from *Bulletin Gen. de Therapeutique*, by F. R. Campbell, M. D.

In spite of the strangeness of this title, we desire to retain it, partly to attract attention, but principally because it expresses very accurately the facts which we wish to describe.

There are frequent cases of clogging of the heart, characterized by a dry, fatiguing cough, and often diagnosticated as muco-purulent bronchial catarrh. The complexus of symptoms observed corresponds very closely with our typical coryza, one of the commonest affections in the world, and one, moreover, of which we know too little of the real pathology. The results obtained by treatment confirm our opinion that there is a "coryza of the heart," and justify our invention of a new word.

We are able to support our theory by arguments drawn from analogy. We state nothing new when we recall the fact that the heart's action is closely related to, and dependent upon, the action of other organs, and that the heart has reciprocally an influence upon the functions of other organs. We are all familiar with the co-existence of hypertrophy of the left ventricle and chronic Bright's disease of the kidneys. We speak of the hepatic heart and a cardiac liver, of stomach cough, uterine cough, etc. It is admitted, moreover, that there are continual reciprocal relations between the functions of the heart and lungs. The heart of asthmatics is often hypertrophied. Those who have heart disease are usually subject to dyspnœa. We may, therefore, easily conceive of a cardiac cough.

For four years, we have treated a lady for an extremely severe recurring attacks of acute coryza, accompanied by marked cardiac disturbance. Although there was no organic disease of the heart, there were great dypnœa, marked exaggeration of the præcordial movements, a congestion of the vessels of the neck—in fact, a disturbance of the entire venous system. There was a complete picture of asystole, brought about by an acute coryza dependent upon meteorological influences. In this case, after employing in vain the remedies usually advised in acute bronchial inflammations, we have invariably given almost immediate relief by using granules of digitalin. In two days, after having taken only four granules of Nativelle digitalin, the coryza assumed its usual benign type, all the cardiac symptoms having disappeared.

In other similar cases, we have attained the same remarkable success in treatment, but we were always of the opinion that our cases were exceptional, and that digitalin would only prove of use in a form of the classical acute catarrh of an essentially cardiac type. But an experience of four years has shown that our idea should be generalized.

It is a well-known fact that a simple "cold" is a very difficult affection to treat. What is the fundamental lesion in a simple cold? A tracheitis, or an inflammation of the larger bronchial tubes? It is a catarrh beginning ordinarily in the nasal fossæ, and descending thence to the pharynx, larynx, trachea, and sometimes reaching even to smaller capillary bronchioles. But these anatomical lesions do not explain some of the most prominent symptoms which characterize the disease. There is an evening exacerbation of temperature, there is an incessant dry cough, there is slight dyspnœa with præcordial anxiety. If the patient remains in bed, the symptoms are not at all alleviated.

Sometimes, this simple "cold" persists for weeks, with no tendency to improvement, and we attribute to neglected "colds" a host of affections.

After the acute symptoms subside, a period of relief comes on. Expectoration is abundant and easy; the patient spits continually, the sputa being of a muco-purulent character, and he often fears the approach of phthisis. To this chain of symptoms, more or less alarming, a routine treatment is directed, or the cold is abandoned to chance, and no attention paid to it. There is a virtual confession on the part of the profession that little can be done for these affections, and the druggists have a wide field for the sale of their nostrums. If a careful study of the nature of a cold should be made by the profession, it would lead to good results.

Coryzas are generally due to a sudden exposure to cold, as is generally admitted by the patients. In some autopsies, certain lesions of the heart are frequently observed: Whitish patches in the visceral pericardium, traces of endocarditis, partial adhesions of the layers of the pericardium, and swelling of the pleura. And notwithstanding the presence of these grave lesions, nothing is remembered in the history of the case which would lead the physician to suspect their presence. From a clinical point of view, to what do these lesions correspond, if not to those of our simple acute coryza? Sometimes, during an attack of coryza, we find indefinite and painful spots in different parts of the chest; sometimes, the pain resembles that of pleurodynia. Do not these pains explain the existence of the thoracic lesions mentioned above; the whitish plaques and the partial adhesions of pericardium and pleura? In connection with these painful spots, there is a præcordial disturbance and a peculiar kind of respiration which persist until expectoration becomes profuse. Yet in this first stage of the disease, auscultation reveals nothing abnormal in bronchial tubes or substance of lung. The condition of the heart at this period is not well known, yet, in many cases, we have been able to detect a dilatation of the left ventricle. It may be thought that this condition of the heart is very difficult to demonstrate, but, by applying the method of "*outlining the heart*," published in *Union Medicale du Nord-Est*, 1887, we can obtain results far more accurate than can be possibly reached by percussion and auscultation. Palpation, however, shows a pulsation stronger than usual, and extending beyond its ordinary limits. Sometimes, at the base a souffle is heard, which may be attributed by some to the fever present at the beginning of the attack. There is, then, a true choking up of the heart, a fact which furnishes the key to the theory which we advance. What takes place in the second stage of coryza we do not think it necessary to record, since it is merely the *denouement* of the scene which we have just described. It is here that the true "cold" begins, that is, the muco-purulent catarrh of the bronchi, after which all becomes more plain, and the condition of the patient greatly improves with the advent of expectoration.

Let us now analyze the treatment to be employed in "colds," that is, of colds accompanied by cardiac plethora and relaxation of the muscular walls of the heart. First, we may stimulate the excretions by employing hot punch, which increases diaphoresis, a practice often followed by good results. The administration of Dover's powder in a warm aromatic infusion, is based upon the same principle. Then we may employ blood-letting, so commonly practiced in the initial stage of fevers in the past, a practice often attended with the best results.

In this same category of remedies, we may place diuretics, purgatives, derivatives, revulsives, etc., all of which are measures calculated to diminish cardiac engorgement and favor contraction of the heart walls when the muscular tone is diminished. We would not advise puncture of the heart, an operation which has several times been practiced (Bruhl, *Progres Med.*, 1887). This operation is only indicated *in extremis*, and even in the worst cases the practice must be dangerous, and attended with only temporary relief.

But the practitioner possesses a powerful weapon for combating this disease in *digitalis*, a true cardiac tonic, which will make him master of the situation.

It is not necessary to enter into any elaborate argument to prove that this is the true therapeutic measure to be adopted in treating our classical "cold" or influenza. Any practitioner has abundant material in his practice to demonstrate the efficacy of this remedy.

Although our first trials were made with Nativelle digitalin, we now prefer preparations of normal digitalis, and especially the tincture of digitalis. In practice, we may combine the tincture of ipecac and opium of the pharmacopœia with our preparation of digitalis; the doses to be used, the time of administration, etc., must be determined by the judgment of the practitioner.

There are now numerous substitutes for digitalis. We may mention, among them, caffeine, sparteine and strophanthine. Caffeine is a remarkable cardiac tonic and diuretic; of sparteine we know but very little; of strophanthine, we know that it acts much like digitalis, arresting, in poisonous doses, the heart in systole. At present, however, we see no occasion for seeking a better remedy than digitalis in the treatment of this affection, called so variously coryzæ, "cold," influenza and acute nasal catarrh.

